# Dialysis modality and mortality of the Contemporary Infant and Neonatal Dialysis (COINED) Cohort: a Pediatric Nephrology Research Consortium (PNRC) study

**DOI:** 10.1007/s00467-025-07082-9

**Published:** 2026-01-13

**Authors:** Melissa Muff-Luett, Tennille Webb, Rebecca Scobell, Neha Pottanat, Eileen Ciccia, Donna Claes, Caitlin Carter, Sarah Nelson-Taylor, Morgan Beebe, Meredith Harris, Issa Alhamoud, Catherine Joseph, Matt Grinsell, Yonique Petgrave, Marc B. Lande, Kelsey Richardson, Bakri Alzarka, Randa Razzouk, Kim Piburn, Rushelle Byfield, Hannah Kim, Chryso Pefkaros Katsoufis, Clare Lindner, Brendan Crawford, Leonela Villegas, Robin High, Elizabeth Lyden, Keia Sanderson

**Affiliations:** 1 Division of Pediatric Nephrology, University of Nebraska Medical School/Children’s Nebraska, 8200 Dodge St, Omaha, NE 68114 USA; 2 Department of Pediatrics, Division of Pediatric Nephrology, University of Alabama at Birmingham/Children’s of Alabama, Birmingham, AL USA; 3 Division of Nephrology, Department of Pediatrics, Children’s Hospital of Philadelphia, Philadelphia, PA USA; 4 Division of Pediatric Nephrology, Department of Pediatrics, Indiana University School of Medicine and Riley Children’s Health, Indianapolis, IN USA; 5 Division of Nephrology, Hypertension, & Apheresis, Department of Pediatrics, Washington University School of Medicine, St. Louis, MO USA; 6 Division of Nephrology, Department of Pediatrics, University of Cincinnati College of Medicine/Cincinnati Children’s Hospital Medical Center, Cincinnati, OH USA; 7 Department of Pediatrics, Division of Pediatric Nephrology, UC San Diego/Rady Children’s Hospital, San Diego, CA USA; 8 Division of Pediatric Nephrology, University of Colorado School of Medicine/Children’s Hospital of Colorado, Aurora, CO USA; 9 Nationwide Children’s Hospital, Columbus, OH USA; 10 Division of Nephrology, Ann & Robert Lurie Children’s Hospital of Chicago, Chicago, IL USA; 11 Division of Pediatric Nephrology, Stead Family Children’s Hospital, University of Iowa, Iowa City, IA USA; 12 Division of Pediatric Nephrology, Baylor College of Medicine, Texas Children’s Hospital, Houston, TX USA; 13 Division of Nephrology and Hypertension, Department of Pediatrics, University of Utah School of Medicine, Salt Lake City, UT USA; 14 Division of Nephrology, Department of Pediatrics, University of Tennessee Health Sciences Center, Memphis, TN USA; 15 Division of Nephrology, Department of Pediatrics, University of Rochester Medical Center, Rochester, NY USA; 16 Department of Pediatrics, Division of Pediatric Nephrology, Oregon Health & Science University, Portland, OR USA; 17 Department of Pediatrics, Division of Pediatric Nephrology, University of Maryland School of Medicine, Baltimore, MD USA; 18 Cook Children’s Medical Center, Fort Worth, TX USA; 19 Division of Nephrology, Department of Pediatrics, The University of Texas Health Science Center at San Antonio, San Antonio, TX USA; 20 Division of Nephrology and Hypertension, Department of Pediatrics, Columbia University Vagelos College of Physicians and Surgeons/ New York Presbyterian-Morgan Stanley Children’s Hospital, New York, NY USA; 21 Division of Nephrology, Penn State College of Medicine/Weill Cornell Medicine, Hershey, PA USA; 22 Division of Pediatric Nephrology, Department of Pediatrics, University of Miami Miller School of Medicine/ Holtz Children’s Hospital, Miami, FL USA; 23 Division of Nephrology, Department of Pediatrics C.S. Mott Children’s Hospital/The University of Michigan, Ann Arbor, MI USA; 24 Division of Nephrology, Department of Pediatrics, University of Arkansas Medical Sciences/Arkansas Children’s Hospital, Little Rock, AR USA; 25 Division of Nephrology, Department of Pediatrics, Connecticut Children’s Medical Center, Hartford, CT USA; 26 University of Nebraska Medical School: Department of Biostatistics, College of Public Health, Omaha, NE USA; 27 University of North Carolina Department of Medicine-Nephrology, Chapel Hill, NC USA

**Keywords:** Neonatal dialysis, Peritoneal dialysis, CKRT, AKI, Stage 5 CKD

## Abstract

**Background:**

Recent technologic advances in neonatal dialysis have changed current dialysis practices. The goal of this study was to define demographics, diagnoses, initial dialysis modality, modality changes, and outcomes of neonates receiving dialysis.

**Methods:**

Retrospective, multicenter cohort of neonates (≤ 30 days of age) from 26 U.S. centers, who received dialysis between 6/2017 and 5/2022. Measures of central tendency were calculated to describe the cohort stratified by the primary dialysis-related diagnosis including acute kidney injury requiring dialysis (AKI-D), stage 5 chronic kidney disease (CKD 5), hyperammonemia, and other causes. The primary outcome was death during the initial hospitalization.

**Results:**

For the 405 neonates in this cohort, AKI-D (57%) was the most common dialysis-related diagnosis, followed by CKD 5 (29%) and hyperammonemia (11%). Most neonates (58%) had a birth weight between 2500 and 3500 g. The most common initial neonatal dialysis modality was continuous kidney replacement therapy (CKRT) with (35%) or without extracorporeal membrane oxygenation (ECMO) (36%). Only 26% of neonates received peritoneal dialysis (PD) as their initial dialysis modality. Thirty percent of neonates received more than one dialysis modality. The overall mortality was 50% (59% in AKI-D and 41% in CKD 5). Withdrawal of care was reported as the most common reason for death. The odds of death were lower for neonates with AKI-D receiving PD as the initial modality when compared to CKRT (aOR 0.28, 95% CI 0.09–0.84) or CKRT with ECMO (aOR 0.24, 95% CI 0.10–0.58), but this association was not seen in neonates with CKD 5.

**Conclusions:**

This multicenter, contemporary neonatal dialysis cohort shows increased use of CKRT as the initial dialysis modality but also shows that the mortality of neonates on dialysis remains high. Additional studies on modern neonatal dialysis are needed.

**Graphical abstract:**

**Figure:**
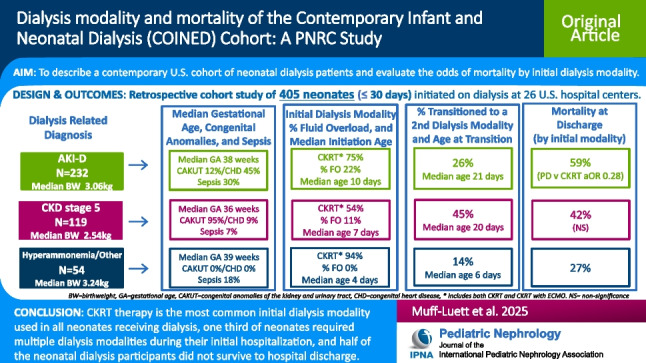
A higher resolution version of the Graphical abstract is available as Supplementary information

**Supplementary Information:**

The online version contains supplementary material available at 10.1007/s00467-025-07082-9.

## Background

United States (U.S.) registry data estimate that the incidence of dialysis for end-stage kidney disease or stage 5 chronic kidney disease (CKD 5) in neonates is approximately 0.3 cases per million live births [1, 2]. Single-center studies estimate that 2–5% of neonates with acute kidney injury (AKI) require dialysis [3–5]. Dialysis use in neonates is rare, technically complex and associated with higher rates of complications including catheter-related complications and infections in comparison to older children receiving dialysis [2, 6, 7]. These complications can lead to discontinuation of dialysis, transition to alternative dialysis modalities, and death.

Despite this, an increasing number of neonates are surviving complex perinatal courses which include dialysis [7]. While previous literature has focused primarily on the use of peritoneal dialysis (PD) for renal replacement therapy in neonates, contemporary advances in dialysis technologies and greater clinical expertise are leading to increased use of CKRT in more medically complex neonates [3, 8–12]. With these advances, an increasing number of neonates are receiving dialysis via modalities other than PD in the neonatal period. The impact of the use of CKRT versus PD in neonates requiring dialysis remains uncertain. Given the rare nature of neonatal dialysis, single-center data are sparse, and more information is needed regarding neonatal dialysis management and outcomes during this time frame [13].

Previous multi-center studies regarding neonatal dialysis are reported from registries, claims databases, or medical records – which are limited by diagnostic coding biases, single modalities (i.e. CKRT or PD alone), and/or a lack of details including dialysis access, initiation characteristics, or dialysis-related complications [3, 7, 10, 13–15]. Thus, more information is needed regarding details on dialysis management and outcomes during the neonatal period.

The primary objective of the Contemporary Infant and Neonatal Dialysis (COINED) study was to describe the demographics, initial dialysis modality, modality transition, dialysis liberation, and patient survival in a contemporary cohort of neonates who received dialysis at ≤ 30 post-natal days stratified by primary dialysis-related diagnosis: AKI requiring dialysis (AKI-D), CKD 5, hyperammonemia, or other/unknown causes. A secondary objective was to evaluate the odds of mortality associated with the initial dialysis modality choice.

## Methods

This is a multicenter retrospective cohort study including 26 hospitals from the U.S. This study was approved by the University of Nebraska Institutional Review Board (IRB) (Protocol #0357–22-EP) as well as each local site IRB. Neonates were included if they received dialysis at ≤ 30 days after birth, between June 1, 2017, and May 31, 2022. Neonates were excluded if they received dialysis for prophylactic fluid management following cardiac surgery in the absence of kidney dysfunction or hyperammonemia.

The study endpoints were death, hospital discharge or transfer, or survival to 90 post-natal days during the initial hospitalization associated with neonatal dialysis initiation.

Each institution entered data from its local electronic medical record (EMR) into a 761-question REDCap survey supported by the University of Nebraska.

### Covariates

The primary dialysis-related diagnosis was retrospectively recorded according to the expertise of the pediatric nephrologist site co-investigator as CKD 5, AKI-D, hyperammonemia, or unknown. Congenital anomalies of the kidney and urinary tract (CAKUT), demographic variables, and clinical perinatal variables were self-reported by pediatric nephrologists. Extra-renal anomalies recorded included congenital diaphragmatic hernia, gastrointestinal anomaly defined as any documented congenital anatomical abnormality of the gastrointestinal system, and congenital heart disease (CHD) defined as any documented congenital heart anomaly in the EMR whether confirmed or unconfirmed by echocardiogram. Demographic variables included gestational age at birth documented per medical record, sex, race, and ethnicity, birthweight, and small for gestational age status. Clinical perinatal variables included 1- and 5-min APGAR scores, other non-renal congenital anomalies, and clinical sepsis, and mechanical ventilation within 72 h prior to dialysis initiation.

Dialysis treatment characteristics, including dates of dialysis catheter placement, dialysis initiation, and dialysis discontinuation were recorded from the EMR. Discontinuation of dialysis was defined by either a period where dialysis was paused due to kidney recovery or for any reason longer than 72 h. When neonates were transitioned between multiple dialysis modalities during their initial hospitalization, dates of dialysis access placement and dialysis initiation, as well as the rationale for dialysis modality changes were recorded.

Percent fluid overload was calculated using the following formula: [closest weight (kilograms (kg)) within 72 h prior to dialysis initiation/birthweight (kg)] × 100 [16].

Medications including vasopressors, diuretics, paralytics, vancomycin, gentamicin, and hydrocortisone were recorded if at least one dose was administered within 72 h prior to dialysis initiation.

### Statistical analyses

Patient demographic data was summarized using counts, percentages, median and interquartile range (IQR) and was compared between primary dialysis-related diagnosis groups using Fisher’s exact test for categorical data and the Kruskal–Wallis test for continuous data. We assessed median birthweight by the primary dialysis-related diagnosis and stratified by mortality. Finally, in a multivariate analysis, we used a random effects logistic regression model with a logit link to evaluate the odds of death based upon the initial dialysis modality for the neonates with AKI-D or CKD 5. The location of the hospital was applied as a random effect to account for correlation due to clustered observations. We adjusted for CHD, fluid overload as a dialysis indication, the presence of mechanical ventilation 72 h prior to dialysis, and sepsis within 72 h prior to dialysis in the logistic regression model for AKI-D. We adjusted for fluid overload as a dialysis indication, the presence of mechanical ventilation 72 h prior to dialysis, and sepsis within 72 h prior to dialysis in the logistic regression model for CKD 5. These adjustments were made based upon prior knowledge of the increased association between death and CHD, sepsis, fluid overload, and respiratory failure [17]. We were unable to adjust for other hypothesized factors that might confound the association between the initial dialysis modality and death due to the rarity of the exposure events in the cohort or due to co-linearity with other variables (i.e. congenital diaphragmatic hernia, and vasopressor exposure) which we originally hypothesized to interact with the exposure of dialysis modality and the outcome of death. Pairwise comparisons of differences in logit means between primary dialysis-related diagnosis groups were adjusted with the simulation technique, the recommended approach for correlated data models [18]. All analyses were completed with the GLIMMIX procedure from SAS/STAT software from Version 9.4 (©2016) of the SAS System for Windows (Cary, NC). Graph figures were produced with the SGPLOT procedure with SAS/BASE software.

## Results

### Participating center demographics

Of the 26 U.S. centers which participated in this study, there were six centers in the Northeast, eight centers in the South, eight centers in the Midwest, and 4 centers in the West per the U.S. Census Bureau definitions of U.S. regions [19]. The median number of pediatric nephrologists practicing in the participating centers was 6.5 (IQR 5,9) with a minimum of 2 and a maximum of 23 physicians and the median number of advanced practice providers per center was 1 (IQR 1,3) with a minimum of 0 and a maximum of 9 advanced practice providers.

### Patient demographics

**Fig. 1:**
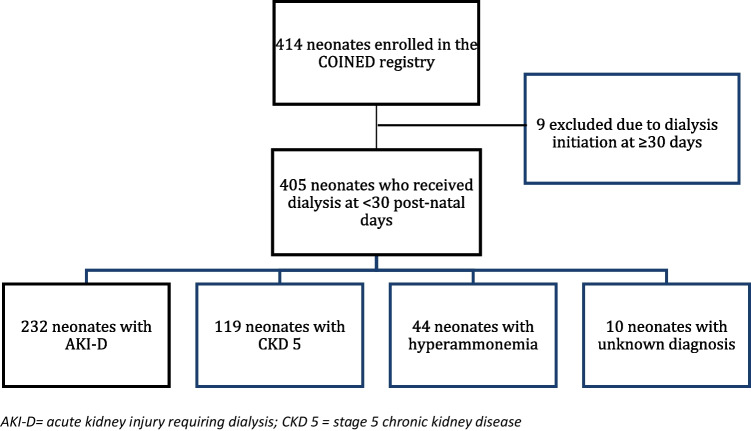
Consort Diagram of COINED registry participants

This study identified 405 neonates who met enrollment criteria and received dialysis at ≤ 30 days of age (Fig. 1). This cohort included 232 neonates (57%) with AKI-D, 119 (29%) with CKD 5, 44 (11%) with hyperammonemia, and 10 (2%) as other or unknown diagnoses. The median gestational age of the cohort was 37 (36,39) weeks. One hundred fifty neonates (37%) were preterm born. Males comprised 60% of the overall cohort which was similar among the dialysis-related diagnoses. Fifty-seven percent of the cohort identified as White race, 17% Black and 16% Hispanic/Latino ethnicity, and this distribution was similar across the study sub-groups. Approximately 58% of the neonates who received dialysis had a birthweight of 2501–3500 g (g), and only 13% of the overall cohort was characterized as small for gestational age (Table 1). Among the neonates with AKI-D, 64% had a birthweight of 2501–3500 g while 45% of neonates with CKD 5 had a birthweight of 2501–3500 g. Interestingly, 46% of neonates with CKD 5 weighed less than 2500 g, but only 16% of neonates with AKI-D weighed less than 2500 g. The difference in the median birthweight between the kidney diagnosis groups specifically, the median birthweight differs between AKI-D and CKD 5 and hyperammonemia and CKD 5 (Fig. 3A). The median 1- and 5-min APGAR scores were 6 and 8, respectively.

**Table 1:** Clinical characteristics of the neonates initiated on dialysis by primary dialysis-related diagnosis

Characteristics	Total N = 405	AKI-D N = 232	CKD 5 N = 119	Hyperammonemia N = 44	Other/Unknown N = 10	p-value
**Gestational age** (wks), median (IQR)	37 (36,39)	38 (36,39)	36 (34,37)	39 (37,39)	38 (36,39)	< 0.0001
**Sex (M)**, n (%)	241 (60)	130 (56)	73 (61)	31 (71)	7 (70)	0.28
**Race,** n (%)						0.877
White	231 (57)	127 (55)	73 (61)	25 (57)	6 (60)	
Black	68 (17)	39 (17)	20 (17)	8 (18)	1 (10)	
Other	21 (5)	11 (5)	6 (5)	3 (7)	1 (10)	
Unknown	85 (21)	55 (24)	20 (17)	8 (18)	2 (20)	
**Ethnicity**, n (%)						0.88
Hispanic/Latino	65 (16)	37 (16)	18 (15)	8 (18)	2 (20)	
**Birth weight,** (g) median (IQR)	2955 (2466,3290)	3062 (2620,3350)	2540 (2200,3020)	3087 (2580,3405)	3397 (3295,3570)	< 0.0001
**Birth weight category**, n (%)	––	––	––	––	––	
< 1000 g	8 (2)	5 (2)	3 (3)	––	––	
1001–1500 g	93 (23)	33 (14)	51 (43)	9 (20)	––	
1501–2500 g	233 (58)	148 (64)	53 (45)	26 (59)	6 (60)	
2501–3500 g	62 (15)	41 (18)	12 (10)	7 (16)	2 (20)	
> 3500 g Unknown	9 (2)	5 (2)	–	2 (5)	2 (20)	
**SGA**, n (%)	52 (13)	32 (14)	13 (11)	7 (16)	–	< 0.0001
**APGARs** md (IQR)						
1 min	6 (3,8)	6 (3,8)	6 (3,7)	8 (8,8)	6 (3,8)	< 0.0001
5 min	8 (6,9)	8 (5,9)	7 (6,8)	9 (9,9)	9 (7,9)	< 0.0001
**CAKUT**, n (%)	140 (35)	27 (12)	113 (95)	–	–	< 0.0001
**Other congenital anomalies**, n (%)						
Congenital heart disease	118 (29)	105 (45)	11 (9)	2 (5)	–	< 0.0001
Congenital diaphragmatic hernia	34 (8)	31 (13)	1 (1)	–	2 (20)	< 0.0001
Gastrointestinal anomaly	20 (5)	10 (4)	8 (7)	1 (2)	1 (10)	0.405
**Sepsis**, n (%)*	91 (22)	70 (30)	8 (7)	8 (18)	5 (50)	< 0.0001
**Mechanical Ventilation**, n (%)*	341 (84)	205 (88)	88 (74)	39 (89)	9 (90)	0.0055
**Medications prior to dialysis Initiation**, n (%)*						
Vasopressors	257 (64)	180 (78)	46 (39)	24 (55)	7 (70)	< 0.0001
Hydrocortisone	178 (44)	125 (54)	37 (31)	12 (27)	4 (40)	< 0.0001
Diuretics	220 (54)	176 (76)	32 (27)	6 (14)	6 (60)	< 0.0001
Vancomycin	73 (18)	62 (27)	5 (4)	5 (11)	1 (10)	< 0.0001
Gentamicin	83 (21)	50 (22)	17 (14)	13 (30)	3 (30)	0.096
**Percent fluid overload,** median (IQR)	12 (0,29) n = 257 (63)	22 (3,41) n = 140 (60)	11 (0, 21) n = 79 (66)	0 (0,0) n = 33 (75)	0 (0,18) n = 5 (50)	< 0.0001

AKI-D = acute kidney injury requiring dialysis, CKD 5 = stage 5 chronic kidney disease; SGA = small for gestational age; CAKUT = congenital anomalies of the kidney and urinary tract; *Occurring or administered at least once within the 72 h prior to dialysis initiation

When examining the underlying kidney diagnoses and other congenital anomalies in this study population, 35% of the neonates had an underlying diagnosis of CAKUT, including 12% of those with AKI-D. Twenty-nine percent of neonates had a CHD, 8% had a congenital diaphragmatic hernia, and 5% had a gastrointestinal anomaly overall. Sepsis was reported in 22% of all participants, and mechanical ventilation was reported in 84% of neonates at the time of dialysis initiation. Sixty-four percent of participants required vasoactive medications and 44% required hydrocortisone within 72 h prior to dialysis initiation. Other congenital anomalies, sepsis, the need for mechanical ventilation from 72 h to dialysis initiation, and all medications recorded within 72 h prior to dialysis were more common in the neonates with AKI-D in comparison to the neonates with CKD 5 (Table 1). Table 1 demonstrates statistically significant differences in the majority of the clinical characteristics in the clinically distinct AKI-D, CKD 5, and hyperammonemia patient populations.

### Dialysis characteristics

**Fig. 2:**
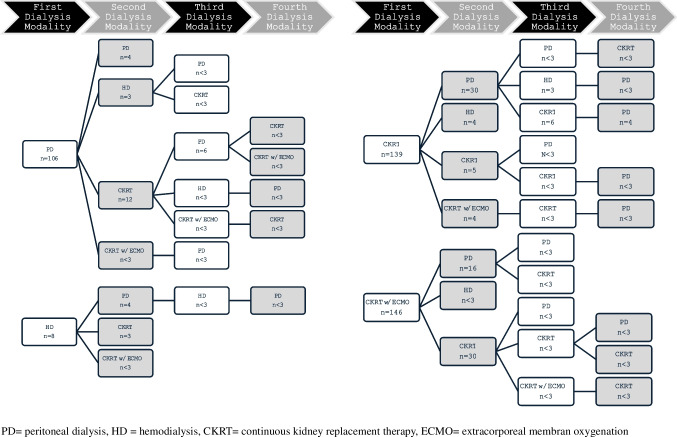
Series of dialysis modality changes that occurred for this cohort by initial dialysis modality

Fluid overload was reported as the most common primary indication for dialysis in 68% of the overall cohort and was common in neonates both with AKI-D (86%) and CKD 5 (55%). However, only 63% (n = 257) of the cohort had a weight reported within 72 h prior to dialysis initiation. Other indications for dialysis initiation included oliguria/anuria (56%), uremia (24%), hyperkalemia (13%) and metabolic acidosis (13%). The average age at dialysis initiation in our neonatal cohort was 9 days which was similar among all the study sub-groups, with the exception of hyperammonemia, in which dialysis was initiated at 4 days (Table 2). Continuous kidney replacement therapy (CKRT) with and without extracorporeal membrane oxygenation (ECMO) was the predominant initial dialysis modalities utilized (35% and 36%, respectively). CKRT with ECMO was the predominant modality for neonates with AKI-D (55%). CKRT and PD were utilized most in neonates with CKD 5 (46% and 43%, respectively), and CKRT was utilized most for those with hyperammonemia (80%). Thirty percent of our cohort experienced one change in dialysis modality, with 9% undergoing a second modality change, and 5% a third modality change. Figure 2 illustrates the series of dialysis modality changes that occurred for this cohort by initial dialysis modality. Dialysis modality changes were more common in neonates with CKD 5 (45%). The most common reason for a dialysis modality change was ECMO decannulation (10%) followed by a dialysis complication (9%), or a planned conversion to peritoneal dialysis from a hemodialysis modality (8%).

**Table 2:** Neonatal dialysis initiation characteristics by primary dialysis related diagnosis

	**Total** **N = 405**	**AKI-D** **N = 232**	**CKD 5** **N = 119**	**Hyperammonemia** **N = 44**	**Other/** **Unknown** **N = 10**
**Age at dialysis initiation**, (days) md (IQR)	9 (4,15)	10 (5,16)	7 (4,14)	4 (3,9.5)	8 (5,11)
**Initial dialysis modality**, n (%)	405	232	119	44	10
PD	106 (26)	54 (23)	51 (43)	1 (2)	–
HD	8 (2)	2 (1)	4 (3)	2 (5)	–
CKRT	143 (35)	46 (20)	55 (46)	35 (80)	7 (70)
CKRT with ECMO	146 (36)	128 (55)	9 (8)	6 (14)	3 (30)
Other	2 (1)	2 (1)	–	–	–
**Rationale for modality change**, n(%)					
Dialysis Complication	35 (9)	18 (8)	16 (13)	1 (2)	–
Planned Conversion to PD	32 (8)	5 (2)	27 (23)	–	–
ECMO Decannulation	39 (10)	30 (13)	7 (6)	2 (5)	–
Other	14 (3)	8 (3)	3 (3)	3 (7)	–
**Age at second dialysis modality**, (days) md (IQR)	20 (12,29)	21 (13,28)	20 (11,43.5)	5.5 (4,14)	–
**Second dialysis modality** n (%)	120 (30)	61 (26)	53 (45)	6 (14)	–
PD	55 (46)	22 (36)	33 (62)	–	
HD	7 (6)	5 (8)	2 (4)	–	
CKRT	51 (42)	30 (49)	17 (32)	4 (67)	
CKRT with ECMO	7 (6)	4 (7)	1 (2)	2 (33)	
**Age at third dialysis modality**, (days) md (IQR)	43 (30,76)	39.5 (30,47)	73 (30.5,85.5)	–	–
**Third dialysis** modality, n (%)	35 (9)	14 (6)	21 (18)	–	–
PD	14 (40)	8 (57)	6 (29)		
HD	5 (14)	1 (7)	4 (19)		
CKRT	14 (40)	4 (29)	10 (48)		
CKRT with ECMO	2 (6)	1 (7)	1 (5)		
**Age at fourth dialysis modality,** (days) md (IQR)	70 (36,111)	36 (32,43)	86.5 (65,116)	–	–
**Fourth dialysis modality**, n (%)	19 (5)	5 (2)	14 (12)	–	–
PD	13 (68)	2 (40)	11 (79)		
HD	–	–	–		
CKRT	5 (26)	3 (60)	2 (14)		
CKRT with ECMO	1 (5)	–	1 (7)		

AKI-D = acute kidney injury requiring dialysis, CKD 5 = stage 5 chronic kidney disease, PD = peritoneal dialysis, HD = hemodialysis, CKRT = continuous kidney replacement therapy, ECMO = extracorporeal membrane oxygenation

### Outcomes

**Fig. 3:**
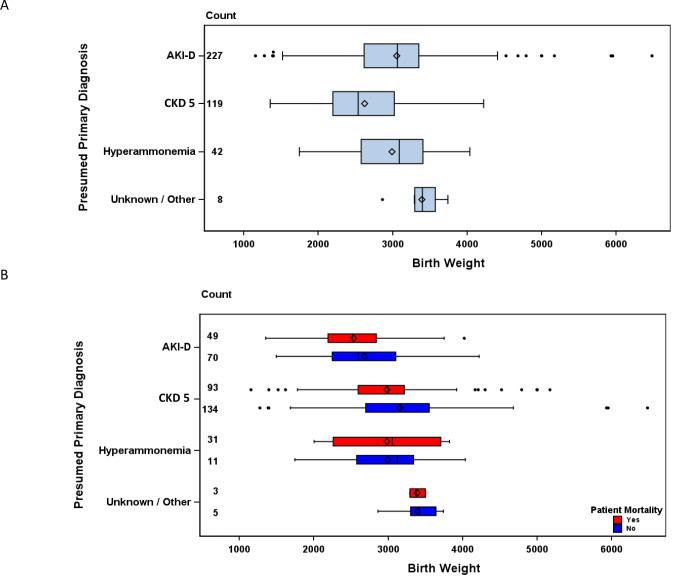
Median birthweight of neonates initiated on dialysis by primary dialysis-related diagnosis (**A**), and by survival status (**B**)

Fifty percent of this neonatal cohort did not survive to hospital discharge and the highest mortality was among neonates with AKI-D (59%) when compared to 42% in CKD 5 (Table 3). There were no differences in survival by birthweight within the primary dialysis-associated diagnoses (Fig. 3B). Withdrawal of care was listed as the most common reason for death across all study sub-groups (63%). Sixty percent of participants died while on dialysis; of those who died, most were on CKRT (49%) or CKRT with ECMO (33%) at the time of death. One hundred sixteen neonates (28%) were able to discontinue dialysis during the initial hospital admission, and 41% remained on dialysis at hospital transfer/discharge at study end. The majority of neonates on dialysis at hospital transfer/discharge were receiving PD (93%) (Table 3).

**Table 3:** Outcomes of neonates initiated on dialysis by primary dialysis-related diagnosis

	**Total** **N = 405**	**AKI-D** **N = 232**	**CKD 5** **N = 119**	**Hyperammonemia** **N = 44**	**Other/Unknown** **N = 10**
**Death during initial admission**, n (%)	203 (50)	138 (59)	50 (42)	12 (27)	3 (30)
**Cause of death, n (%)**					
Withdrawal of care	128 (63)	91 (66)	26 (52)	8 (67)	3 (100)
Respiratory failure	16 (8)	12 (9)	4 (8)	–	–
Cardiac failure	30 (15)	24 (17)	6 (12)	–	–
Infection	11 (5)	6 (4)	3 (6)	2 (17)	–
Other/Unknown	18 (9)	5 (4)	11 (22)	2 (17)	–
**Receiving dialysis at time of death**, n (%)	121/203 (60)	77/138 (56)	33/50 (66)	9/12 (75)	2/3 (67)
**Dialysis modality at time of death, n (%)**					
PD	19 (16)	13 (17)	6 (18)	–	–
CKRT	59 (49)	22 (29)	26 (79)	9 (100)	2 (100)
CKRT with ECMO	40 (33)	39 (51)	–	–	–
Missing	3 (2)	3 (4)	1 (3)	–	–
**Liberated from dialysis prior to transfer/discharge**, n (%)*	116/198 (59)	72/91 (79)	5/68 (7)	32 (100)	7 (100)
**Transferred/discharged on dialysis**, n (%)	82/198 (41)	19/91 (21)	63/68 (93)	–	–
**Dialysis modality at transfer/discharge**, n (%)					
PD	75/82 (93)	18/19 (95)	57/63 (92)	–	–
HD	5/82 (6)	1/19 (5)	4/63 (6)	–	–
Unknown	2/82 (1)	–	2/63 (2)	–	–

^*^4 participants missing whom survived to time of study end but remained hospitalized at time of study end

In the logistic regression analysis, the odds of death were lower for neonates with AKI-D initially receiving PD in comparison to neonates initially receiving CKRT (aOR 0.28, 95% CI 0.09–0.84) and CKRT with ECMO (aOR 0.24, 95% CI 0.10–0.58). There were no significant differences in mortality among neonates with AKI-D initially receiving CKRT vs. CKRT with ECMO, nor were there significant differences in mortality by initial modality for neonates with CKD 5 (Tables 4 and 5).

**Table 4:** Mortality by initial dialysis modality of neonates with acute kidney injury requiring dialysis (AKI-D)

Characteristics	Adjusted OR*	95%CI	p-value
PD vs CKRT	0.28	0.09–0.84	0.02
PD vs CKRT ECMO	0.24	0.10 −0.58	< 0.001
CKRT vs CKRT ECMO	0.86	0.34–2.19	0.92

AKI-D adjusted for congenital heart disease, fluid overload as an indication for dialysis initiation, mechanical ventilation within 72 h prior to dialysis initiation, and sepsis within 72 h prior to dialysis initiation

**Table 5:** Mortality by initial dialysis modality of neonates with stage 5 chronic kidney disease (CKD 5)

Characteristics	Adjusted OR*	95%CI	p-value
PD vs CKRT	0.69	0.16 −3.00	0.82
PD vs CKRT ECMO	0.40	0.04–3.62	0.58
CKRT vs CKRT ECMO	0.58	0.08–4.29	0.79

Adjusted for fluid overload as an indication for dialysis initiation, mechanical ventilation within 72 h prior to dialysis initiation, and sepsis within 72 h prior to dialysis initiation

## Discussion

In this multicenter cohort, we describe the demographics, clinical characteristics, and initial dialysis modalities within a large U.S. cohort of neonates with CKD 5, AKI-D, or hyperammonemia. Regardless of the underlying dialysis-related diagnosis, the most common initial modality for all neonates in this contemporary cohort from 2017 to 2022 was CKRT with or without ECMO, while only one quarter of neonates received PD as a first dialysis modality. Overall, the mortality of neonates receiving dialysis was 50% during the initial hospitalization. Among neonates with AKI-D, receiving CKRT as the initial modality was associated with 72% greater odds of death in comparison to neonates who received PD first, even when adjusting for factors associated with a greater severity of illness. The implications of this research demonstrate that there is a significant shift in the practice of neonatal dialysis away from PD in the U.S. for both acute and chronic dialysis, but the impact of this on patient survival is unclear.

Several previously published multicenter studies of infants and neonates with CKD 5 have primarily utilized claims data from EMR databases, the United States Renal Data Systems (USRDS), or registries that include neonates on dialysis who have already survived to hospital discharge or are limited by the details of dialysis access and modality characteristics [6, 10, 14, 20]. While PD has been the most common modality reported for neonates with CKD 5, only 40% of neonates with CKD 5 received PD as their initial dialysis modality in the COINED cohort. This is far less than previously published data, despite PD being considered the preferred modality for neonates with CKD 5 [3, 8–10]. This indicates a significant change in dialysis practice patterns for neonates with CKD 5 in the U.S. with an increasing use of CKRT as the initial modality. Despite this change in practice, nearly all neonates with CKD 5 who survived to hospital discharge/transfer in the COINED cohort were receiving PD at the time.

The median birthweight of the COINED cohort was similar to previously published multicenter neonatal dialysis cohorts, with most neonates weighing 2500–3500 g [10, 13, 14]. Although there are single-center studies and case reports of neonates successfully receiving dialysis at less than 1000 g, there were no neonates in this cohort with birthweights < 1000 g who were initiated on dialysis; and fewer than 10 neonates were initiated on dialysis with birthweights < 1500 g. This suggests that currently in the U.S. very low birthweight neonates are rarely initiated on dialysis. Moreover, in the COINED cohort, there were no differences between the birthweight of neonates who survived versus those who did not survive (Fig. 3B). We believe this is a result of low variation in birthweight in this cohort and a lack of any extremely low birthweight infants for comparison. Interestingly, despite the consistent report of birthweights for the whole cohort, only 63% of the cohort had a weight measured and reported within the 72 h prior to dialysis initiation. This highlights what is likely a common challenge for neonates receiving dialysis which may benefit from continuous quality improvement.

Studies of neonates with AKI-D have focused almost exclusively on CKRT, in comparison to this cohort which includes the evaluation of neonates receiving multiple dialysis modalities. Several studies have examined CKRT in neonates and infants with AKI-D, including modified Aquapheresis therapy and Carpediem™ [11, 12, 15, 21, 22]. Most recently, the WE-ROCK cohort described 75 infants less than 5 kg and 47 neonates < 1 month of age with AKI or fluid overload who received therapy with only CKRT [23]. The COINED cohort also demonstrates that while the majority of neonates with AKI-D received CKRT as an initial modality, over one quarter transitioned to a second modality during their initial hospitalization, most often to PD. We think this reflects a common practice pattern of CKRT initiation in anticipation of immediate dialysis needs which allows transition to a well-healed PD catheter exit site. Unfortunately, CKRT and CKRT with ECMO as initial dialysis modalities in the AKI-D subpopulation were associated with a higher risk of death in comparison to PD. Previously published neonatal dialysis research has not compared outcomes between neonates with AKI-D who initially received PD versus those who received CKRT. Factors associated with PD versus CKRT choice as the initial modality for neonates with AKI-D deserve further study to help elucidate neonatal dialysis practice changes and to evaluate for unmeasured factors which may contribute to greater mortality.

The COINED cohort demonstrates that one third of all neonates initiated on dialysis underwent at least one dialysis modality change during the first 90 days of life. Claes et al. similarly reported 23.4% of their cohort received PD plus another extracorporeal dialysis modality prior to death or hospital discharge [13]. The reason for this trend is unclear but likely reflects the difficulty of neonatal dialysis and the high risk for complications, which likely require changes in modality. Long- term studies are needed to evaluate the impact on vessel preservation and future dialysis access for neonates who undergo multiple dialysis modality changes within the first months of life.

Finally, while it is known that the survival rate of infants receiving dialysis is lower than that of older children receiving dialysis, more recent studies show improving survival trends in neonates receiving dialysis upwards of 70–80% at hospital discharge and 1 year following dialysis initiation [8, 10, 13–15]. The survival rate in this COINED cohort was much lower than these reports, with an overall survival rate at hospital transfer/discharge of 50%. The lowest survival rate occurred in neonates with AKI-D (41%), likely due to a greater severity of illness within this cohort. This is supported by a high incidence of CHD and sepsis among the neonates with AKI-D. Mortality rates were similar between the COINED AKI-D cohort and the WE-ROCK cohort, which reported a mortality rate of 55% in the 47 neonates. Prior neonatal dialysis research has a lower incidence of neonates who required CKRT with ECMO, suggesting that poorer survival within the COINED cohort may be due to neonates with a greater severity of illness [6, 10, 14, 15, 22, 24]. The survival of neonates with CKD 5 in the COINED cohort was higher than that of neonates with AKI-D. The survival rate of 58% in the COINED CKD 5 cohort differs from Claes et al., as well as other CKD 5 registry cohorts which have reported survival rates of 76–80% at hospital discharge for neonates born with CKD 5 [6, 10, 13]. The reason for this increased mortality rate in neonates with CKD 5 in the COINED cohort is uncertain but may be a reflection of a smaller and more medically complex population of neonates with CKD 5 who are now receiving dialysis. However, this research challenges the benefits of CKRT versus PD use in the neonatal CKD 5 population and is a call to action for additional research to improve dialysis therapies for neonates.

While dialysis in neonates is a relatively rare occurrence, the number of participants and centers within the COINED cohort increases the generalizability of this study’s results. Study limitations include that the data collection method required manual database entry from an EMR, which could potentially result in center selection bias and data entry errors. This study did not include data on neonates who may not have been offered dialysis based on their size, gestational age, underlying diagnosis, co-morbidities, or severity of illness. Though we provided guidance for identifying eligible subjects at each center, we cannot guarantee all eligible neonatal dialysis patients were enrolled from every center. Each center self-defined the primary dialysis-related diagnoses of AKI-D, CKD 5, hyperammonemia, and other, and the dialysis initiation indications per their center criteria and pediatric nephrology expertise. This may have contributed to misclassification bias. However, most of the surviving neonates classified as CKD 5 remained on dialysis at study end (96%) and in comparison, few surviving neonates with a diagnosis of AKI-D remained on replacement therapy (3%) at study end, an outcome that is consistent with what would be expected of patients classified as CKD 5 and AKI-D. There are also unmeasured factors contributing to each neonate’s dialysis course, including additional medications, details on changes to the patient’s fluid status, and additional co-morbidities. These factors may contribute to competing risks for mortality after dialysis initiation. Finally, withdrawal of care was reported as the most common reason for death in this COINED cohort and no other details were available regarding this variable. Future research should focus on factors associated with withdrawal of care among neonates initiated on dialysis.

## Conclusions

The COINED study is a modern neonatal cohort showing a major shift in dialysis practices within the U.S. CKRT was the most common initial dialysis modality for neonates with AKI-D and CKD 5, not PD. Neonatal dialysis often involves multiple dialysis modality changes prior to hospital discharge. However, most neonates requiring dialysis at hospital discharge are receiving PD. In addition, dialysis is not commonly offered to neonates weighing less than 1500 g in the U.S and most importantly, the mortality of the neonates in this study receiving dialysis is higher than previously reported. While we cannot establish causality, it is possible that sicker neonates are receiving dialysis therapies in the current era owing to advances in the devices available for neonatal dialysis. Regardless, this data indicates that, in the AKI-D neonatal population, the use of PD as an initial modality was associated with a 72% reduction in the odds of mortality in comparison to CKRT and highlights the need for additional research on neonatal dialysis modalities including those that highlight the complications and outcomes of neonates receiving PD as compared to infants receiving CKRT for AKI and CKD 5.

## Supplementary Information

Below is the link to the electronic supplementary material.

Graphical abstract (PPTX 87.3 KB)

## Data Availability

Deidentified individual data that supports the results will be shared beginning 12 to 36 months following publication provided the investigator who proposes to use the data has approval from an Institutional Review Board (IRB), Independent Ethics Committee (IEC), or Research Ethics Board (REB), as applicable, and executes a data use/sharing agreement with the University of Nebraska. When data sharing is permitted, a Data Use Agreement (DUA) specifying the uses of such data to be shared must be in place before any data is shared. Requests can be submitted to the Principal Investigator, Dr. Melissa Muff-Luett for processing. The Principal Investigator must confirm that the DUA has been fully executed, and IRB, IEC, or REB approval has been granted before sharing data.
