# Supplementary material for: Dialysis modality and mortality of the Contemporary Infant and Neonatal Dialysis (COINED) Cohort: a Pediatric Nephrology Research Consortium (PNRC) study
Source: Pediatr Nephrol. 2026 Jan 13;41(6):1731–42. doi: 10.1007/s00467-025-07082-9 (PMC13139246; doi:10.1007/s00467-025-07082-9)
Supplement: Supplementary file 1 — Graphical abstract (PPTX 87.3 KB) [file 467_2025_7082_MOESM1_ESM.pptx]

## Slide 1
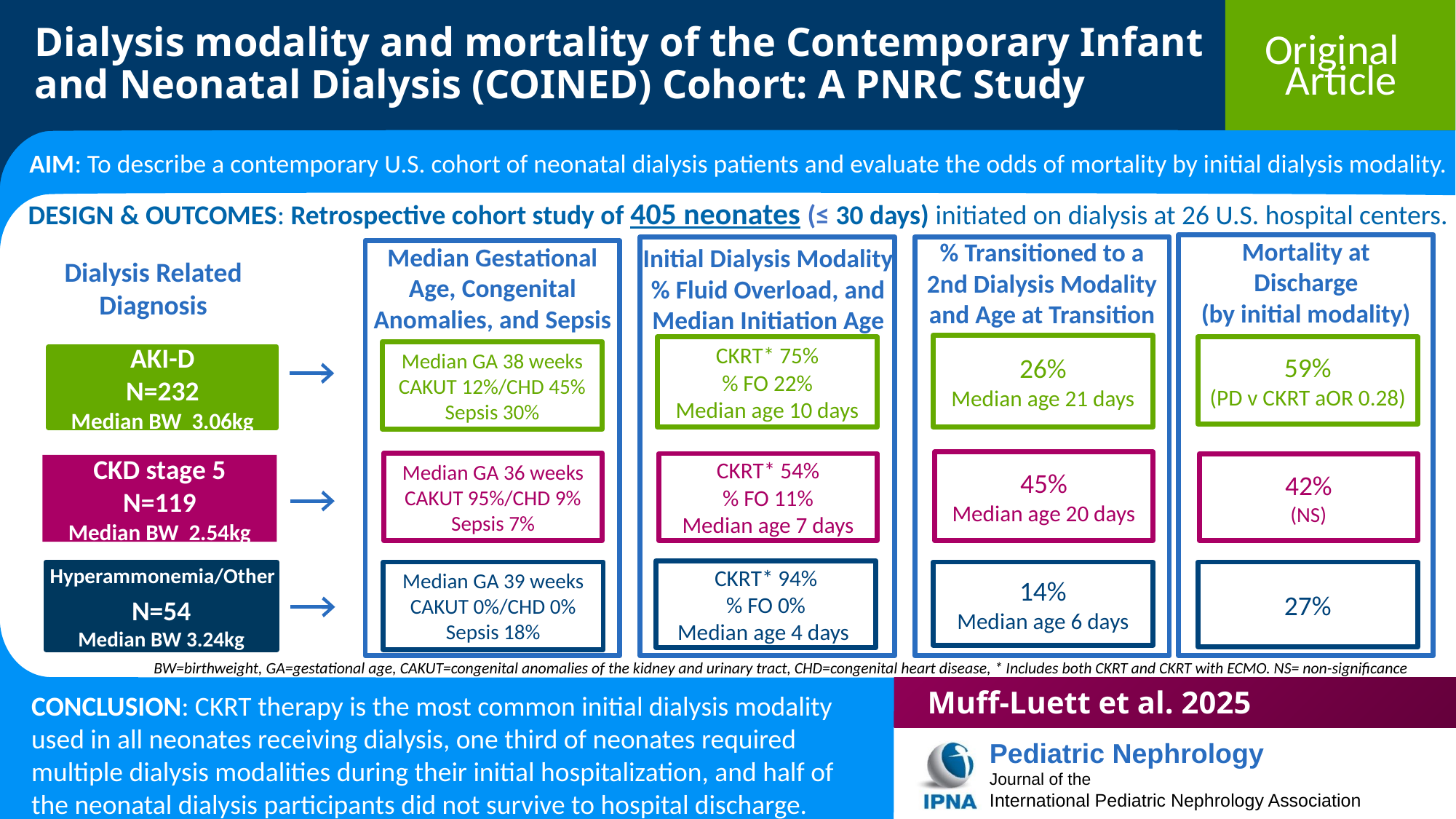

Dialysis modality and mortality of the Contemporary Infant and Neonatal Dialysis (COINED) Cohort: A PNRC Study
AIM: To describe a contemporary U.S. cohort of neonatal dialysis patients and evaluate the odds of mortality by initial dialysis modality.
DESIGN & OUTCOMES: Retrospective cohort study of 405 neonates (≤ 30 days) initiated on dialysis at 26 U.S. hospital centers.
Mortality at Discharge
(by initial modality)
Median Gestational Age, Congenital Anomalies, and Sepsis
Median GA 38 weeks
CAKUT 12%/CHD 45%
Sepsis 30%
Median GA 36 weeks
CAKUT 95%/CHD 9%
Sepsis 7%
Median GA 39 weeks CAKUT 0%/CHD 0%
Sepsis 18%
Initial Dialysis Modality
% Fluid Overload, and Median Initiation Age
% Transitioned to a 2nd Dialysis Modality and Age at Transition
Dialysis Related Diagnosis
26%
Median age 21 days
CKRT* 75%
% FO 22%
Median age 10 days
59%
(PD v CKRT aOR 0.28)
AKI-D
N=232
Median BW 3.06kg
45%
Median age 20 days
42%
(NS)
CKRT* 54%
% FO 11%
Median age 7 days
CKD stage 5
N=119
Median BW 2.54kg
Hyperammonemia/Other
CKRT* 94%
% FO 0%
Median age 4 days
N=54
Median BW 3.24kg
14%
Median age 6 days
27%
BW=birthweight, GA=gestational age, CAKUT=congenital anomalies of the kidney and urinary tract, CHD=congenital heart disease, * Includes both CKRT and CKRT with ECMO. NS= non-significance
Muff-Luett et al. 2025
CONCLUSION: CKRT therapy is the most common initial dialysis modality used in all neonates receiving dialysis, one third of neonates required multiple dialysis modalities during their initial hospitalization, and half of the neonatal dialysis participants did not survive to hospital discharge.
